# Supplementary material for: Fluorescent organelle markers in Cryptococcus neoformans: a versatile toolkit for live-cell subcellular localization
Source: bioRxiv. 2026 Mar 4:2026.03.03.709387. Preprint. [Version 1] doi: 10.64898/2026.03.03.709387 (PMC13001312; doi:10.64898/2026.03.03.709387)
Supplement: Supplement 1 — Figure S1. Construction and functional validation of mCherry labeled organelle marker. a) Genotype validation of mCherry-tagged organelle marker strains. Correct integration of the mCherry tagging cassette at the safe haven locus was verified by diagnostic PCR, confirming both 5’-end and 3’-end homologous recombination events as illustrated in the schematic shown in Fig. 1b. All strains shown carry SH:PH3–gene–mCherry integrations, with individual genes encoding organelle markers fused to mCherry at the safe haven locus. The corresponding organelles and strain identifiers are indicated: NOP1 (nucleolus, YSB11829), DCP1 (P-body, YSB11825), DNJ1 (ER, YSB11832), KTR3 (Golgi, YSC8), CAP6 (Golgi, YSC11), VCX1 (vacuole, YSB11822), RAB5 (endosome, YSC9), MJR1 (mitochondria, YSB11818), ANT1 (peroxisome, YSB11817), ATG8 (autophagosome, YSB11835), and PMA1 (plasma membrane, YSC4). “mCh” indicates the mCherry-only control strain (YSC1), while WT and DW represent the H99 wild-type strain and distilled water control, respectively. b) Gene expression analysis of genes flanking the safe haven locus and mCherry-tagged target genes. Transcript levels of genes located upstream and downstream of the safe haven integration site, as well as the corresponding mCherry-tagged target genes, were quantified by qRT-PCR. Expression levels were compared between the H99 wild-type strain (WT) and strains carrying SH:PH3-gene-mCherry-HYG integrations. Data are shown as relative expression normalized to WT. Bars represent the mean ± SEM. Statistical significance was assessed using one-way ANOVA with Bonferroni’s multiple-comparison test (ns, not significant; ***, p < 0.001; ****, p < 0.0001). c) Stress susceptibility assay. The susceptibility of the wild-type (WT) strain and mCherry-tagged cellular marker strains to antifungal drugs and other stress-inducing agents was examined. Cells were cultured overnight in YPD medium at 30°C, serially diluted (10-fold), and spotted onto YPD agar plates contain [file media-1.pdf]

Figure S1 (Choi et al.)

a

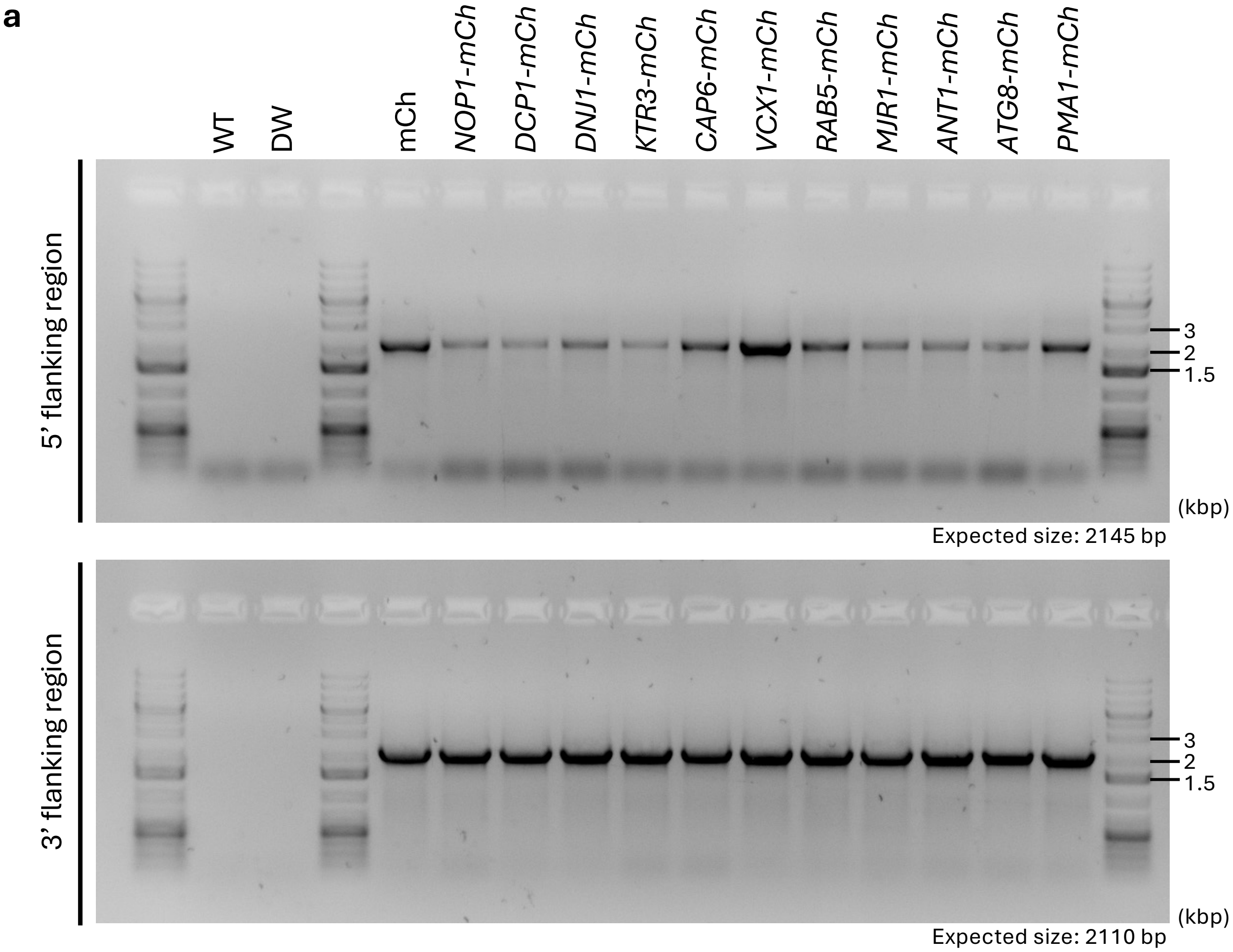

Figure S1a. Genotype validation of mCherry-tagged organelle marker strains.

Figure S1 (Choi et al.)

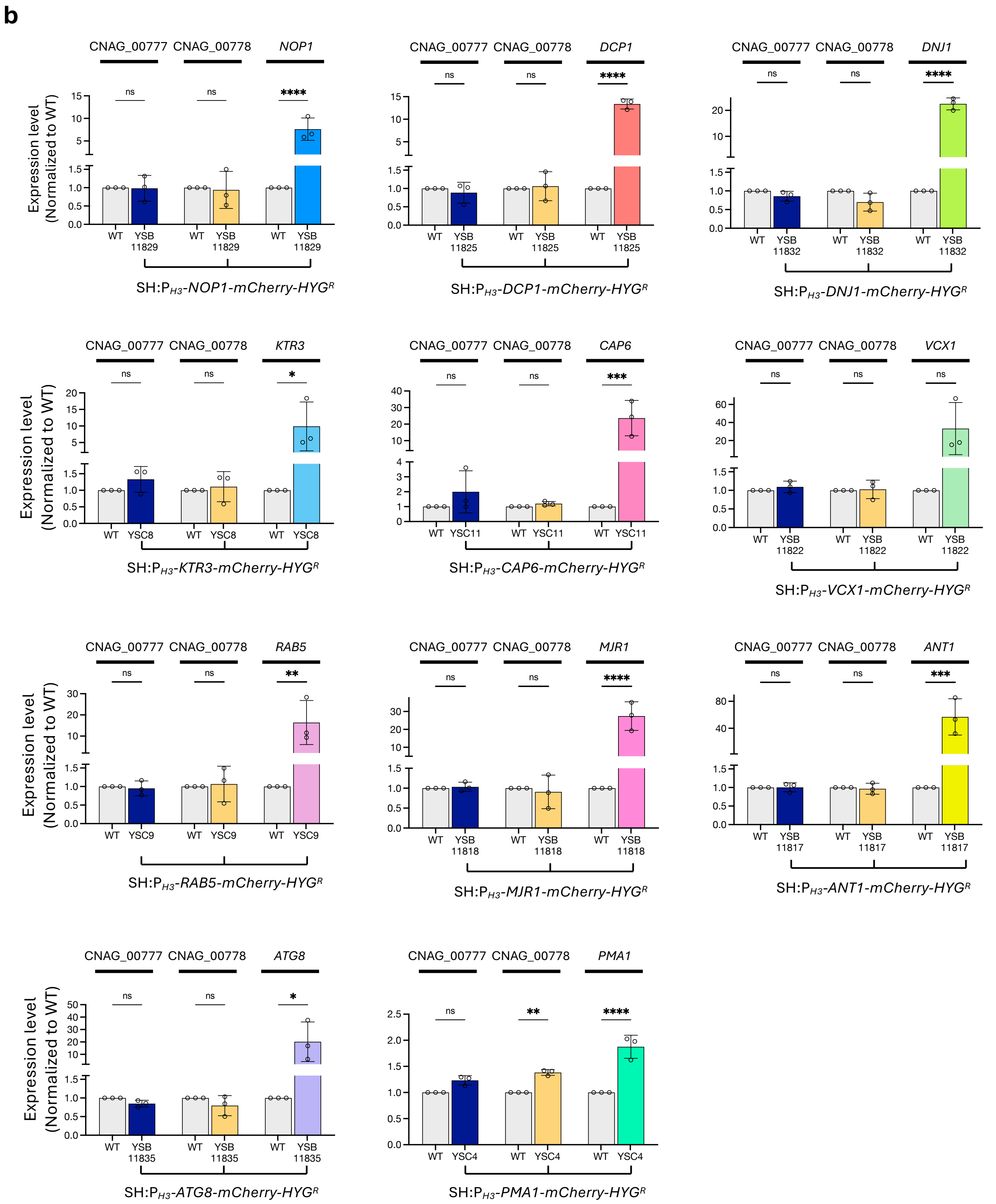

Figure S1b. Gene expression analysis of genes flanking the safe haven locus and mCherry-tagged target genes.

Figure S1 (Choi et al.)

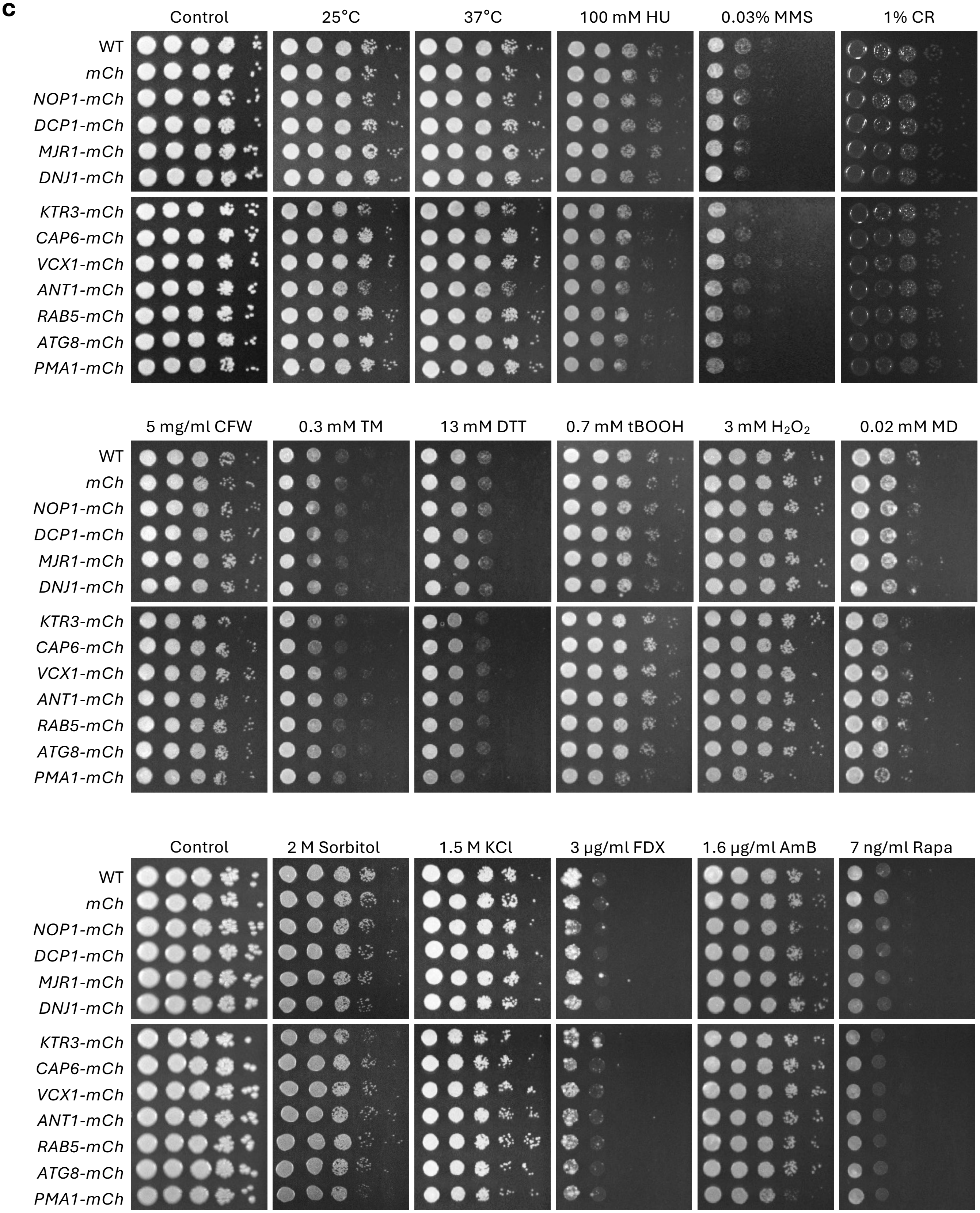

Figure S1c. Stress susceptibility assay.

Figure S2. (Choi et al.)

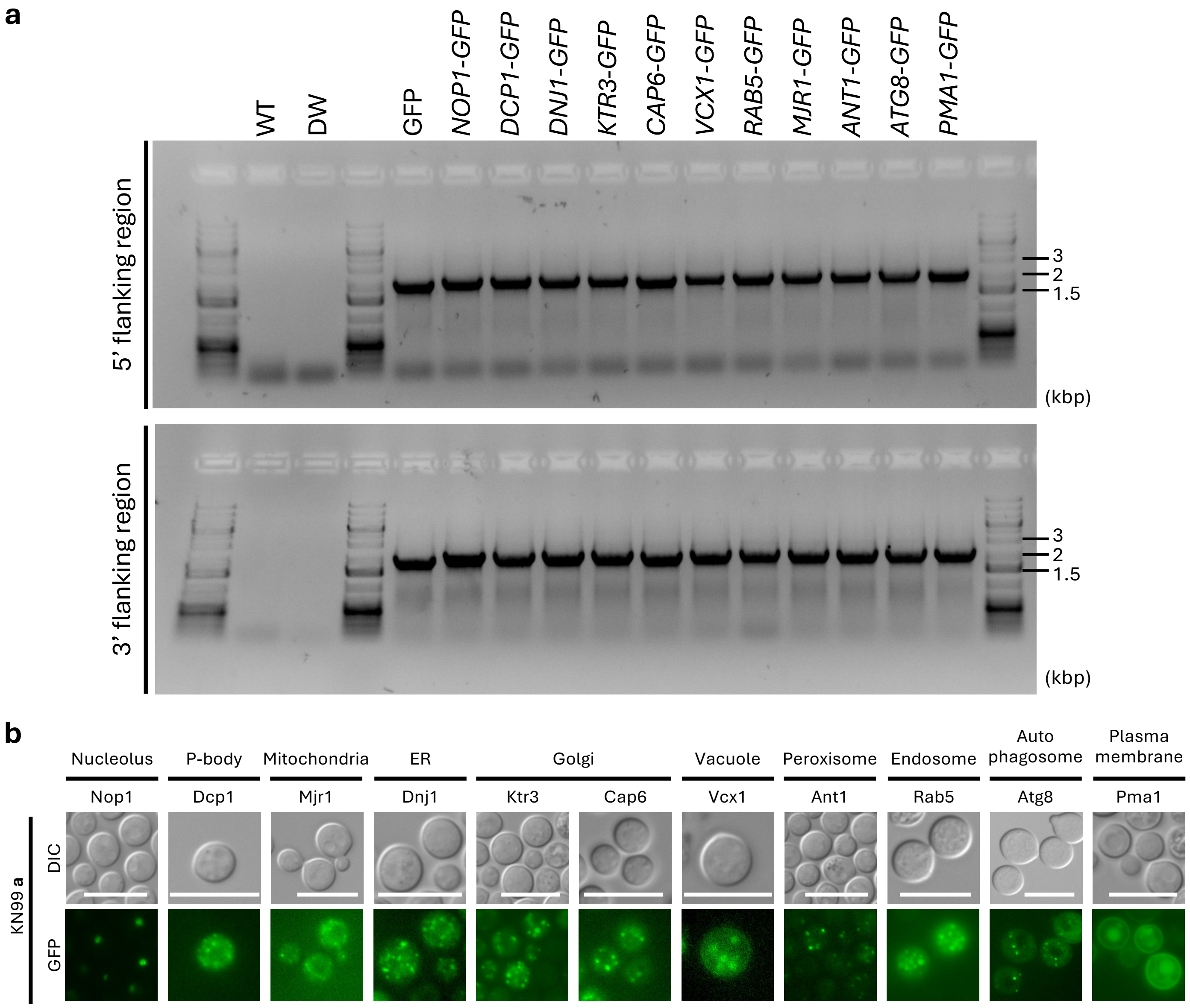

Figure S2. Construction of GFP-tagged cellular marker strains.
